# First Molecular Characterisation of Porcine Parvovirus 7 (PPV7) in Italy

**DOI:** 10.3390/v16060932

**Published:** 2024-06-08

**Authors:** Silvia Dei Giudici, Lorena Mura, Piero Bonelli, Luca Ferretti, Salwa Hawko, Giulia Franzoni, Pier Paolo Angioi, Anna Ladu, Graziella Puggioni, Elisabetta Antuofermo, Maria Luisa Sanna, Giovanni Pietro Burrai, Annalisa Oggiano

**Affiliations:** 1 Department of Animal Health, Istituto Zooprofilattico Sperimentale della Sardegna, 07100 Sassari, Italyannalisa.oggiano@izs-sardegna.it (A.O.); 2 Nuffield Department of Medicine, Big Data Institute and Pandemic Sciences Institute, University of Oxford, Oxford OX1 4BH, UK; 3 Department of Veterinary Medicine, University of Sassari, 07100 Sassari, Italy

**Keywords:** parvovirus, pigs, PPV7, Italy

## Abstract

Porcine parvoviruses (PPVs) are among the most important agents of reproductive failure in swine worldwide. PPVs comprise eight genetically different species ascribed to four genera: *Protoparvovirus* (PPV1, PPV8), *Tetraparvovirus* (PPV2-3), *Copiparvovirus* (PPV4-6), and *Chaphamaparvovirus* (PPV7). In 2016, PPV7 was firstly detected in the USA and afterwards in Europe, Asia, and South America. Recently, it was also identified in Italy in pig farms with reproductive failure. This study aimed to evaluate the circulation of PPV7 in domestic and wild pigs in Sardinia, Italy. In addition, its coinfection with Porcine Circovirus 2 (PCV2) and 3 (PCV3) was analysed, and PPV7 Italian strains were molecularly characterised. PPV7 was detected in domestic pigs and, for the first time, wild pigs in Italy. The PPV7 viral genome was detected in 20.59% of domestic and wild pig samples. PPV7 detection was significantly lower in domestic pigs, with higher PCV2/PCV3 co-infection rates observed in PPV7-positive than in PPV7-negative domestic pigs. Molecular characterisation of the NS1 gene showed a very high frequency of recombination that could presumably promote virus spreading.

## 1. Introduction

Parvoviruses are non-enveloped, small, single-stranded, linear DNA viruses of the family Parvoviridae [1]. These viruses have an icosahedral capsid containing a genome of 4–6.3 kb in length. According to the actual virus classification compiled by the International Committee on the Taxonomy of Viruses (ICTV), Parvoviruses are subdivided into three subfamilies. These three groups are Parvovirinae, causing infection in vertebrates; Densovirinae, infecting arthropods; and Hamaparvovirinae, which are able to infect both invertebrates and vertebrates. Porcine parvoviruses (PPVs) include viruses that infect swine, comprising the genera Protoparvovirus (PPV1, PPV8), Tetraparvovirus (PPV2-3), Copiparvovirus (PPV4-6), and Chaphamaparvovirus (PPV7) of the subfamily Hamaparvovirinae [2,3,4,5]. PPV1 was isolated from cell culture in Germany in 1965. PPV2 to PPV6 were detected progressively between 2001 and 2014 in several countries, mainly the USA and China [6,7,8], whereas PPV7 was recently discovered in 2016 in rectal swabs of apparently healthy pigs in America [9]. Analogously to other PPVs, the PPV7 genome consists of two major open reading frames (ORFs), ORF1 and ORF2. ORF1 encodes the non-structural protein 1 (NS1), which is responsible for the replication of the virus. ORF2 encodes the capsid protein (Cap), also considered the most immunogenic component in PPVs [9,10,11]. The similarity in the amino acids (aa) of the NS1 between PPV7 and other parvoviruses is less than 30%, leading to its assignment to a new genus [9]. PPV1 is globally considered one of the major agents of reproductive failure in swine, causing recurring oestrus, abortion, and the delivery of stillborn or mummified foetuses (SMEDI: stillbirth, mummification, embryonic death, and infertility) [12,13]. PPV7 was also detected in aborted pig foetuses and pig semen, suggesting its involvement/role in reproductive failures and its possible transmission through artificial insemination [14,15]. It was also detected in combination with Porcine Circovirus 2 (PCV2) and Porcine Circovirus 3 (PCV3) in sows with reproductive dysfunction [16,17]. Co-infections are quite common in swine herds, as reported by other studies, where concurrent infections of PPVs, including PPV7, with PCVs in pigs were observed [18]. Still, PPV7’s pathogenicity and its role as a co-factor in PCV-associated diseases enhancing lesion severity remains to be clarified [19,20]. PPV7 has been detected in various tissue samples (serum, stool, aborted foetuses, nasal swabs, liver, lung, kidney, spleen, and lymph nodes) in both healthy and unhealthy pigs [11,21,22]. Nonetheless, limited knowledge regarding PPV7’s role in causing disease is available and it is debated whether PPV2-8 can be considered commensal without any significant impact on pig health [23,24]. The presence of PPV7 was reported in several regions worldwide, such as in North and South America [25,26,27], China [10,11,28], the Republic of Korea [29,30], and Australia [31]. In Europe, PPV7 was firstly detected in Poland [32] and subsequently in Sweden [33]. More recently, the presence of the newly emerging PPV7, together with other PPV species, was revealed in Northern Italy in pig farms experiencing reproductive failure [34], but no information about the molecular characteristics of the strains was reported. Hence, this study was performed on blood and tissue samples collected from Sardinian domestic and wild pigs to investigate the presence of PPV7, to molecularly characterise the Sardinian strains and perform a time-scaled phylogenetic analysis. Co-infection with PCV2 and PCV3 was also analysed.

## 2. Materials and Methods

### 2.1. Sample Collection

In this study, 311 samples (131 blood sera and 180 tissues) from domestic and wild swine were collected between 2020 and 2022 and were analysed at the Diagnostic Virology Laboratory of Istituto Zooprofilattico Sperimentale (IZS) of Sardinia:

- 189 samples (87 blood sera and 102 tissue samples, among which 76 had a history of reproductive failure) from domestic pigs throughout the Sardinian regional territory;
- 86 samples (44 blood sera and 42 spleens) from hunted wild boars collected during the 2020–2022 hunting seasons in north Sardinia (Sassari province);
- 36 spleen samples obtained from feral free-ranging pigs culled between 2021 and 2022 in central Sardinia (Nuoro province), during the depopulation action of the Sardinian African Swine Fever Virus (ASFV) eradication plan.

### 2.2. DNA Isolation, PPV7, PCV2, and PCV3 Detection

Tissue samples were homogenised in PBS at 10% (*w*/*v*). Viral DNA was then extracted from either serum or homogenised tissue samples using the MagMax Core kit and MagMax96 extractor (Thermo Fisher, Waltham, MA, USA), according to the manufacturer’s instructions. Samples were stored at −80 °C until further analysis.

All samples were screened for the presence of PPV7, PCV2, and PCV3 by qualitative real-time PCR, using primers previously described [9,35,36]. Samples with a Ct value of less than 40 were considered positive. 

### 2.3. PPV7 ORF1 Sequencing

Nine PPV7 positive samples with Ct values < 30 were selected for sequencing. The complete ORF1 sequence of PPV7 encoding for the NS1 protein was obtained via amplification of three overlapping amplicons produced by PCR using primers reported in Table 1. 

In detail, amplicons were separated using 2% agarose gel and subsequently purified using the QIAquick Gel Extraction Kit (Qiagen, Hilden, Germany), according to the manufacturer’s instructions. Purified samples were stored at −20 °C until further analysis.

Sequencing was performed using a DNA sequencing kit (dRhodamine Terminator Cycle Sequencing Ready Reaction; Thermo Fisher, Waltham, MA, USA) on an ABI-PRISM 3500 Genetic Analyzer (Thermo Fisher, Waltham, MA, USA). BioEdit software version 7.0.0 [37] was used to perform editing, assembling, and translation of the sequences. BLAST nucleotide analysis (https://blast.ncbi.nlm.nih.gov accessed on 26 January 2024) was performed for their identification. Haplotypes of sequences were identified using DNASPv6 [38].

The complete NS1 region of nine Sardinian strains was aligned with all NS1 sequences available on GenBank (accessed 31st October 2023), which were both published in international journals and with a collection date specified. The final dataset, named dataset1, was composed of 61 sequences, including international sequences (*n* = 52) and Italian PPV7 strains obtained in this study (*n* = 9). Dataset1 was checked for recombination by RDP, GENECONV, MaxChi, and 3Seq methods in the RDP5 software v.5.52 [39]. To confirm the results obtained in RDP5, detected recombinant strains were further analysed in SimPlot software v.3.5.1 [40]. The evolutionary model that best fitted the data was selected using JmodelTest v.2.1.7 [41]. Furthermore, the NS1 sequences included in dataset1 were split into three non-overlapping contiguous regions, using the criterion that the cumulative genetic diversity in each region (as characterised by the number of SNPs in the alignment) would be a third of the overall cumulative genetic diversity. Dataset2 comprised the first 588 nucleotides, dataset3 nucleotides 589–1260, and dataset4 the last 759 nucleotides. Datasets2–4 were checked for recombination, as already described above for dataset1.

### 2.4. Phylogenetic Analysis

The phylogenetic signal of the four datasets was subjected to likelihood mapping analysis, with 10,000 random quartets in the TreePuzzle software v.5.2 [42]. 

Phylogeny was estimated in MEGA 7 [43] via the maximum likelihood and the GTR + G + I model of nucleotide substitution pre-estimated. Statistical support for specific clades was obtained via 1000 bootstrap replicates. The time-scaled phylogenetic reconstruction was performed in BEAST software v.1.10.4, using the GTR + G + I model of nucleotide substitution and the Bayesian Markov Chain Monte Carlo (MCMC) approach [44].

To investigate the demographic history of datasets2–4, three independent MCMC runs were carried out using a strict, uncorrelated lognormal relaxed clock or a random local clock, and one of the following coalescent priors: constant population size, exponential growth, or non-parametric Bayesian skyline plot. The best-fitting models were selected calculating the marginal likelihood via the path sampling and stepping-stone analysis performed in BEAST v.1.10.4. [45]. The Bayesian maximum clade credibility tree was obtained on datasets2–4 using the clock and the demographic model, previously estimated on dataset1. Chains were carried out for at least 100 × 10^6^ generations and sampled every 10,000 steps. The three independent MCMC runs were combined using LogCombiner v.1.10.4., distributed as part of the BEAST package. Convergence of the MCMC method was assessed by calculating the Effective Sample Size for each parameter in Tracer software v.1.7.2 [46]. Only parameters estimated with ESSs > 200 were accepted. Uncertainty in the estimates was indicated by 95% highest posterior density (95% HPD) intervals. Maximum clade credibility trees were constructed from tree posterior distributions in the Tree-Annotator software v.1.10.4, available in the BEAST package and visualized in FigTree software v. 1.4.4 (http://tree.bio.ed.ac.uk/software/figtree/ accessed on 26 January 2024).

### 2.5. Data Analysis and Statistics

Statistical analyses were carried out using SPSS v.21 software. A Chi-squared test (χ^2^) or a Fisher exact test was used to test differences in the PPV7 detection rate between domestic, free-ranging pigs, and wild boars and to evaluate co-infection with PCV2 and PCV3. *p* values < 0.05 were regarded as statistically significant.

## 3. Results

### 3.1. PPV7 Presence in Sardinian Domestic Pigs and Wild Populations

The PPV7 viral genome was detected in 64 out of 311 samples (20.59%, Table 2). Among the 189 samples from domestic pigs, 3.17% (6/189) were positive for PPV7. Among the samples from wild boars and free-ranging pigs, 37.21% (32/86) and 72.22% (26/36) were positive for PPV7, respectively (Table 2). 

The frequency of PPV7 detection was significantly lower in domestic pigs than in wild boars (Χ^2^ = 57.49, *p* = 0.00001) and free-ranging pigs (Χ^2^ = 111.19, *p* = 0.00001) and in wild boars compared to free-ranging pigs (Χ^2^ = 12.47, *p* = 0.00041). The same significant differences in the three animal groups were found in both serum and tissue samples (Table 2).

### 3.2. PPV7 Co-Infection with PCV2 and PCV3

The real-time PCR for detection of PCV2 and PCV3 evidenced that 156 out of 311 samples (50.16%) were positive for PCV2, and 88 out of 311 (28.29%) for PCV3. Sixty-one out of 311 samples (19.61%) were positive for both viruses (Table 3).

As for PPV7, significant differences were detected in PCV2, PCV3, and PCV2-PCV3 infection rates between the three animal groups (Table 3). Higher detection rates were evidenced in free-ranging pigs than in wild boars or domestic pigs, and in wild boars with respect to domestic pigs. Table 4 reports the infection rates of PCV2, PCV3, or PCV2-PCV3 in samples positive (PPV7+) or negative for PPV7 (PPV7−). 

Among domestic pigs, PPV7-positive samples presented higher rates of positivity for PCV2 and PCV3 with respect to PPV7-negative samples. In particular, 33.33% of PPV7-positive domestic pigs (2 out of 6) were co-infected with both PCV2 and PCV3, whereas only 4.37% (8 out of 183) of PPV7-negative samples presented the viral genome of both PCV2 and PCV3. As shown in Table 4, this difference was statistically significant (Fisher exact test = 0.0338). On the contrary, even if high co-infection rates were observed in the two wild populations, no significant differences were evidenced in the rate of co-infection with PCV2, PCV3, or both viruses in PPV7-positive and PPV7-negative wild boar and free-ranging pig samples (Table 4).

### 3.3. Sequencing and Phylogenetic Analysis

The determined ORF1 sequences of the nine Sardinian PPV7 strains were deposited in GenBank under the following accession numbers: PP472466-PP472474. Detailed information is presented in Table 5.

The NS1 gene sequences were 2019 bp long and showed 93.4–98.9% similarity between themselves and 94.3–95.5% similarity with the reference PPV7 strain, NC040562. Nine different haplotypes were evidenced. Figure S1 shows the amino acid alignment between the sequences obtained in this study and the reference strain NC040562 (China 2015), particularly highlighting the replication initiation motifs (aa 76–83; 100–106; 154–163), NTP-binding motifs (aa 319–326; 361–366), and the helicase domain (aa 397–405), which are necessary for parvovirus replication [47]. Overall, 112 aminoacidic substitutions were detected, three of which were in the replication initiation motifs and one in the helicase domain. The BLAST analysis showed that the Italian sequences had the highest similarity (95.59–99.31%) with strains from China, the USA, and Poland collected in 2016–2022.

The recombinant analysis performed using RDP5 showed a high level of recombination between the sequences included in dataset1. After removing the recombinant sequences detected by more than three methods, the analysis was performed two more times, until no evidence of recombination was found in the alignment. Figure S2 shows the different RDP5 outputs and compatibility matrices (Robinson-Foulds) after the first (Figure S2a) and the following (Figure S2b,c) analyses. A total of 49 out of 61 recombinant sequences were detected by RDP5 software, including six Italian sequences (397/Italy|2020, 422/Italy|2020, 80282-15/Italy|202, 2040/Italy|2020, 2230/Italy|2020, and 63170/Italy|2022). However, the compatibility matrix, which is useful for visualising the overall phylogenetic impacts of recombination in a sequence alignment, still showed residual recombinant signals in the last alignment composed of 12 sequences (Figure S2d). The results obtained using RDP5 were confirmed using SimPlot software.

Considering such a high recombination rate, the whole NS1 sequences were divided into three parts in order to obtain the recombinant-free derived datasets2–4, as confirmed by RDP5 analysis. Independent phylogenetic and time-scaled analyses were performed on datasets2–4.

**Figure 1:**
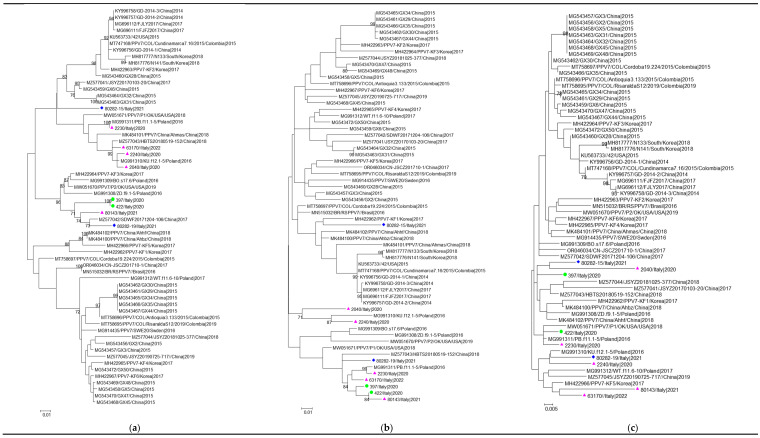
A maximum likelihood phylogenetic tree inferred from datasets 2 (**a**), 3 (**b**), and 4 (**c**) composed of 61 PPV7 strains by the GTR + G + I model of nucleotide substitution. Isolates under study are indicated with different symbols. Pink triangles: wild boar; green circles: domestic pigs; blue diamonds: free-ranging pigs. Bootstrap values < 70 are not shown. The scale bar indicates the number of substitutions per site.

The phylogenetic signals, shown in Figure S3, evidenced sufficient phylogenetic information in all the datasets analysed. The maximum likelihood (ML) phylogenetic trees obtained from the dataset1 and datasets2–4 are shown in Figure S4 and Figure 1, respectively.

The ML tree built on dataset2 placed eight out of nine Italian sequences in three well-supported clusters. Four sequences from two pigs, a wild boar, and a free-ranging pig, coming from three different Sardinian provinces, formed a first separate cluster strongly supported by a high bootstrap of 96%, with sequences from the USA (MW051670; 2019), China (MH422964; 2017), the Republic of Korea (MZ577042; 2017), and Poland (MG991308, MG 991309; 2016). Three Sardinian PPV7 strains from wild boars clustered together (bootstrap 99%), with sequences from China collected in 2018 (MK484101) and Poland (MG991310) isolated in 2016. A third cluster (bootstrap 100%) grouped the strain 2230/Italy|2020 with just one sequence of Polish origin (MG991311; 2016). Sequence 80282-15/Italy|2021 from a free-ranging pig was part of an unresolved cluster (polytomy) grouping strains from different geographical areas.

The ML phylogenetic tree obtained from dataset3 placed seven out of nine Italian sequences in two supported clusters. The first cluster (bootstrap 84%) was composed of Italian sequences from wild boars (n = 3) and domestic pigs (n = 2), together with one sequence from Poland (MG991311, 2016). The second cluster (bootstrap 71%) was composed of two Italian sequences from wild boars and one of Polish origin (MG991310, 2016). Sequences 80282-15/Italy|2021 and 80282-19/Italy|2021 from free-ranging pigs were part of an unresolved cluster grouping strains from different countries. However, the tree inferred from dataset4 showed a different topology and the Italian sequences did not group in any resolved cluster. The results of the path sampling and stepping-stone analysis performed on datasets2–4 showed that the uncorrelated lognormal relaxed clock fitted significantly better, and that the exponential growth model was better than the other models (Table S1). Figure S5 presents the time-scaled tree calculated from datasets2–4. 

The root of the trees estimated on datasets2–4 dates back to the year 1951,39 for dataset2 (95% HPD: 1867,92–1993,64; posterior probability (PP) = 1), 1995,8 (95% HPD: 1961,35–2009,35; PP = 1) for dataset3 and 2011,44 (95% HPD: 2008,9–2013,3; PP = 1) for dataset4. The estimated mutation rates related to the datasets2–4 are presented in Table S2.

Time to the most recent common ancestor (tMRCA) for the Italian strains obtained from datasets2–4 is shown in Table 6. 

## 4. Discussion

This work described PPV7 infection in domestic pigs and, for the first time, its presence in wild pigs in Italy. A study conducted by Miłek et al. in Poland [32], provided the first evidence of detection of PPV7 in domestic pigs in Europe, followed by the report by Blomström et al. [33] in Sweden. More recently, Faustini et al. [34] reported the circulation of PPV7 in domestic pig farms, with reproductive problems in northern Italy in association with other pathogens. 

PPV7 was detected in domestic pigs, as well as in free-ranging pigs and wild boars, even though marked differences in detection rates were evidenced. Indeed, the detection rate in domestic pigs was found to be significantly lower (3.17%) than in free-ranging pigs (72.22%) and wild boars (27.30%). Possibly, the biosecurity measures adopted in domestic pig farms might have played a relevant role in reducing the introduction and spreading of infectious agents, including PPV7. The proportion of PPV7 infection found in this study was similar to that previously reported in Italy (5.9%) [34], but appeared to be lower than in Poland (19.6%) [32], Southern China (25.73%) [28], Mongolia (28.72%) [10], and the Republic of Korea (14.2%) [48]. Few reports on PPV7 infection in wild boars have been published so far; the positivity percentages found in wild boars in the present work are higher than what was observed by other authors in the Republic of Korea (less than 0.5%) [29]. Similarly to what was reported for PPV7, lower PCV2 and PCV3 detection rates were also found in domestic pigs in comparison with free-ranging pigs and wild boars. As already stated [49], these discrepancies between observed infection rates might reflect the different immunological status of domestic and wild pigs induced by an intensive pig production system and the preventive measures adopted. The concurrent infection of domestic and wild pigs with PPV7, PCV2, and PCV3 was also evidenced. PCV2 and PCV2/PCV3 infection rates were found to be significantly higher in PPV7-positive than in PPV7-negative domestic pigs. These findings are in agreement with those of other researchers that have hypothesised PPV7’s contribution to increasing PCV2 viraemia and triggering PCV3 replication [16,19,50]. On the other hand, in wild pigs, PCV2 and PCV3 infection was found at similar percentages in both PPV7-positive and PPV7-negative animals.

In the present work, nine complete NS1 sequences from samples of two domestic pigs, two free-ranging pigs, and five wild boars collected in Sardinia (Italy) were obtained. NS1 is a phosphorylated protein that plays a fundamental role in parvovirus replication, gene expression regulation, host cell apoptosis, and cell cycle arrest [51]. PPV7 NS1 contains ATPase and a helicase domain, which are the only regions exhibiting clear homology to those found in other parvovirus groups [52]. 

Alignment of our sequences with the reference strain (NC040562) evidenced high numbers of nucleotide and amino acid substitutions. Aminoacidic polymorphisms were interestingly detected also in the replication initiation motifs and the helicase domain. NS1 is thought to evolve under selective pressure, as supported by its high mutation rate, which represents the virus strategy to evade the immune response [53,54]. The BLAST analysis found low similarity when comparing these sequences with strains from other geographic areas. As already observed by Milek et al. [32], European PPV7 strains seemed to be more diverse than samples from the USA and China.

Surprisingly, RDP5 and SimPlot analyses of dataset1, including sequences from Italy and other countries, evidenced a high recombination rate (with recombinant fragments observed in 80% of the sequences) that was not previously reported by other authors. Similar recombination rates (77%) were also obtained analysing a dataset composed of a larger number of sequences (n = 174), suggesting that high recombination rates represent a constitutive feature of PPV7 NS1 molecular evolution. Considering these issues, we decided to split the entire length of the NS1 sequence into three parts, and to perform phylogenetic and time-scaled analyses on the derived recombinant-free datasets. 

In the ML tree calculated on datasets2 and 3, most of the Italian sequences were arranged in two main clusters. The ML tree built on dataset*2*, confirming the BLAST analyses results, showed the presence of three well-supported clusters, including Italian sequences from this study and strains from China, Poland, and the USA. However, in dataset3, Italian sequences clustered significantly only with strains from Poland. No statistical support for the Italian strains was found in dataset4.

The time-scaled phylogenetic analysis suggested the occurrence of at least six introduction events of PPV7 in Sardinia, between 1982 and 2019, presumably originating from other countries passing through mainland Italy. The evolutionary rates calculated on the three datasets (Table S2) are in agreement with the estimated mean values previously reported by other authors [14,53]. Furthermore, our analysis evidenced that the root of the trees could be dated back to a span of time from 1951 to 2011. Using a different dataset composed of full genome sequences, including strains collected in 2015–2018 coming mostly from Asia, Wang et al. determined the root in 2004 [53].

In conclusion, our work has evidenced PPV7 circulation in Italian domestic and wild pig populations, with relevant presence in wild animals and PPV7/PCV2/PCV3 co-infection rates in domestic pigs. Molecular characterisation of the NS1 gene showed a very high frequency of recombination that could presumably represent an important impact on virus spreading and transmission dynamics.

## Figures and Tables

**Table 1:** Details of primers used for the sequencing of PPV7 ORF1.

Primer	Sequence	Reference
PPV7-1F PPV7-1R	5′-GCAGCCGCTTCCTGGTGAG-3’ 5′-CCAGRTCGGGYGCGTTTC-3’	This study
PPV7-2F PPV7-2R	5′-CAGGGAGCCTGATGGAATAC-3’ 5′-CTGGATCTGCGAACGAA-3’	[9]
PPV7-3F PPV7-3R	5′-ATCATCATGACGACCAACCACGCAC-3’ 5′-AGGCGCTTTATTGATCACCGAAGC-3’	[11]

**Table 2:** PPV7 infection rate of samples analysed in this study.

Sample	Domestic Pigs Infection Rate (%)	Wild Boars Infection Rate (%)	Free-Ranging Pigs Infection Rate (%)	Total Infection Rate (%)
Blood serum	4/87 (4.6) ^a^	12/44 (27.3) ^b^	/	16/131 (12.21)
Tissues	2/102 (1.96) ^a^	20/42 (47.62) ^b^	26/36 (72.22) ^c^	48/180 (26.67)
Total	6/189 (3.17) ^a^	32/86 (37.21) ^b^	26/36 (72.22) ^c^	64/311 (20.59%)

A Chi-squared test (Χ^2^) was performed between infection rate values of domestic pigs, free-ranging pigs, and wild boars. Different superscripts (^a, b^ or ^c^) within the rows indicate significant differences between the three groups (*p* < 0.05). Identical superscripts (^a, b^, or ^c^) within the rows indicate no significant differences between the three groups (*p* > 0.05).

**Table 3:** PCV2 and PCV3 infection rate of samples analysed in this study.

Sample	PCV2 Infection Rate (%)	PCV3 Infection Rate (%)	PCV2/PCV3 Infection Rate (%)
Domestic pigs	61/189 (32.27) ^a^	30/189 (15.87) ^a^	10/189 (5.29) ^a^
Wild boar	61/86 (70.93) ^b^	31/86 (36.04) ^b^	23/86 (26.74) ^b^
Free-ranging pigs	34/36 (94.44) ^c^	28/36 (77.78) ^c^	28/36 (77.77) ^c^
Total	156/311(50.16)	88/311 (28.29)	61/311 (19.61)

A Chi-squared test (Χ^2^) was performed between PCV2, PCV3, and PCV2/PCV3 infection rate values of domestic pigs, free-ranging pigs, and wild boars. Different superscripts (^a, b^, or ^c^) within the columns indicate significant differences between the three groups (*p* < 0.05). Identical superscripts (^a, b^, or ^c^) within the columns indicate no significant differences between the three groups (*p* > 0.05).

**Table 4:** Infection rates of PCV2, PCV3, and PCV2-PCV3 in PPV7-positive (PPV7+) or PPV7-negative (PPV7−) samples.

		Proportion of Samples (%)	PCV2 Infection Rate (%)	PCV3 Infection Rate (%)	PCV2-PCV3 Infection Rate (%)
Domestic pigs	PPV7+	6/189 (3.17%)	5/6 ^a^ (83.33%)	3/6 ^a^ (50%)	2/6 ^a^ (33.33%)
	PPV7−	183/189 (96.83%)	56/183 ^b^(30.60%)	27/183 ^a^ (14.75%)	8/183 ^b^ (4.37%)
Wild boars	PPV7+	32/86 (37.21%)	23/32 ^a^ (71.87%)	14/32 ^a^ (43.75%)	10/32 ^a^ (31.25%)
	PPV7−	54/86 (62.79%)	38/54 ^a^ (70.37%)	17/54 ^a^ (31.48%)	13/54 ^a^ (24.07%)
Free-ranging pigs	PPV7+	26/36 (72.22)	25/26 ^a^ (96.15%)	21/26 ^a^ (80.77%)	21/26 ^a^ (80.77%)
	PPV7−	10/36 (27.78%)	9/10 ^a^ (90%)	7/10 ^a^ (70%)	7/10 ^a^ (70%)

A Chi-squared (Χ^2^) or Fisher exact test was performed between PCV2, PCV3, or PCV2-PCV3 infection rate values of domestic pigs, free-ranging pigs, and wild boars. Different superscripts within the columns (^a, b^) indicate significant differences between the three groups (*p* < 0.05). Identical superscripts (^a, b^) within the columns indicate no significant differences between the three groups (*p* > 0.05).

**Table 5:** The year of collection, host, municipalities, BEAST IDs, and GenBank accession numbers of PPV7 strains analysed in this study.

Strain	Year	Host	Municipality/Province	BEAST ID	Accession Number
397/Italy|2020	2020	Domestic pig	S. Gavino Monreale (SU)	53IT@20	PP472466
422/Italy|2020	2020	Domestic pig	S. Gavino Monreale (SU)	54IT@20	PP472467
80282-15/Italy|2021	2021	Free-ranging pig	Talana (NU)	55IT@21	PP472468
80282-19/Italy|2021	2021	Free-ranging pig	Talana (NU)	56IT@21	PP472469
2040/Italy|2020	2020	Wild boar	Muros (SS)	57IT@20	PP472470
2230/Italy|2020	2020	Wild boar	Erula (SS)	58IT@20	PP472471
2240/Italy|2020	2020	Wild boar	Tergu (SS)	59IT@20	PP472472
80143/Italy|2021	2021	Wild boar	Illorai (SS)	60IT@21	PP472473
63170/Italy|2022	2022	Wild boar	Padru (SS)	61IT@22	PP472474

**Table 6:** tMRCA, 95%HPD, and PP estimated for datasets2–4. PP > 0.9 in bold characters.

	Dataset2				Dataset3		Dataset4			
BEAST ID	Strain	tMRCA	95% HPD	PP	tMRCA	95% HPD	PP	tMRCA	95% HPD	PP
53IT@20	397/Italy|2020	2019,39	1966,00–2019,50	**1**	2010,06	1997,75–2014,8	**0** **.** **99**	2015,42	2013,21–2017,65	0.33
54IT@20	422/Italy|2020	2019,39	1966,00–2019,50	**1**	2016,69	2011,62–2019,90	**1**	2015,73	2014,23–2017,40	0.32
55IT@21	80282-15/Italy|2021	1984,04	1933,03–2000,60	**0.94**	2012,83	2000,52–2019,32	**0.98**	2015,42	2013,21–2017,65	0.33
56IT@21	80282-19/Italy|2021	2010,87	1995,74–2016,68	0.9	2012,83	2000,52–2019,32	**0.98**	2016,08	2014,15–2018,28	0.54
57IT@20	2040/Italy|2020	2013,72	2007,28–2015,99	**0.96**	2007,08	1989,52–2014-10	0.27	2014,94	2013,33–2016,68	0.02
58IT@20	2230/Italy|2020	2015,04	1977,00–2015,90	**1**	2014,9	2011,37–2016,00	**1**	2016,08	2014,15–2018,28	0.54
59IT@20	2240/Italy|2020	2014,80	1970,00–2015,89	**0.96**	2011,17	2001,00–2015,36	**0.93**	2015,10	1981,00–2015,20	0.65
60IT@21	80143/Italy|2021	2005,4	1981,64–2015,88	0.78	2016,69	2011,62–2019,90	**1**	2016,25	2013,44–2016,68	0.29
61IT@22	63170/Italy|2022	2008,45	1993,38–2014,76	**0.99**	2010,77	1999,45–2014,94	0.55	2015,50	2013,62–2017,09	0.18

In dataset2 and dataset3, Sardinian sequences clustered significantly in different branches of the trees, suggesting that different introduction events of the virus from other countries could have occurred. Instead, in dataset 4, no significant clades containing Italian sequences were found.

## Data Availability

The original contributions presented in the study are included in the article/Supplementary Materials, further inquiries can be directed to the corresponding author/s.

## Supplementary Materials

The following supporting information can be downloaded at https://www.mdpi.com/article/10.3390/v16060932/s1, Figure S1: Amino acid alignment of the Italian PPV7 NS1 sequences with the NC040562 reference strain. The three replication initiation motifs (aa 76–83; 100–106; 154–163), the two NTP-binding motifs (aa 319–326; 361–366), and the helicase domain (aa 397–405), necessary for parvovirus replication, are evidenced in red, green, and blue squares, respectively. Figure S2: RDP4 output and compatibility matrix (Robinson-Foulds) of the alignment composed of 61 sequences (a), 33 sequences (b), 16 sequences (c), and the compatibility matrix of the alignment composed of 12 sequences (d). Figure S3: Phylogenetic signal of the datasets analysed in this study. a: dataset1; b: dataset2; c: dataset3; and d: dataset4. Figure S4: A maximum likelihood phylogenetic tree inferred from dataset1 composed of 61 PPV7 strains by the GTR + G + I model of nucleotide substitution. Isolates under study are indicated with different symbols. Red triangles: wild boar; green circles: domestic pigs; and blue diamonds: free-ranging pigs. Bootstrap values < 70 are not shown. The scale bar indicates the number of substitutions per site. Figure S5: The Bayesian maximum clade credibility tree of dataset 2 (a), 3 (b), and 4 (c). The node age and statistical support for the clade (posterior probability) are shown near the nodes and above the branches, respectively. The scale at the bottom of the tree represents time in years. Table S1: Marginal Likelihood estimates for different molecular clock models of PPV7 datasets2–4 using a Path Sampling and Stepping-Stone analysis (a). Marginal Likelihood estimates for different coalescent models under Uncorrelated lognormal relaxed clock of PPV7 datasets2–4 using a Path Sampling and Stepping-Stone analysis (b). Table S2: Identity, estimated mutation rates (95%HPD), and root dates (95%HPD) for datasets2–4 analysed in this study.
