# Supplementary material for: First Molecular Characterisation of Porcine Parvovirus 7 (PPV7) in Italy
Source: Viruses. 2024 Jun 8;16(6):932. doi: 10.3390/v16060932 (PMC11209580; doi:10.3390/v16060932)
Supplement: Supplementary file 1 [file viruses-16-00932-s001.zip › Figure S1.pptx]

## Slide 1
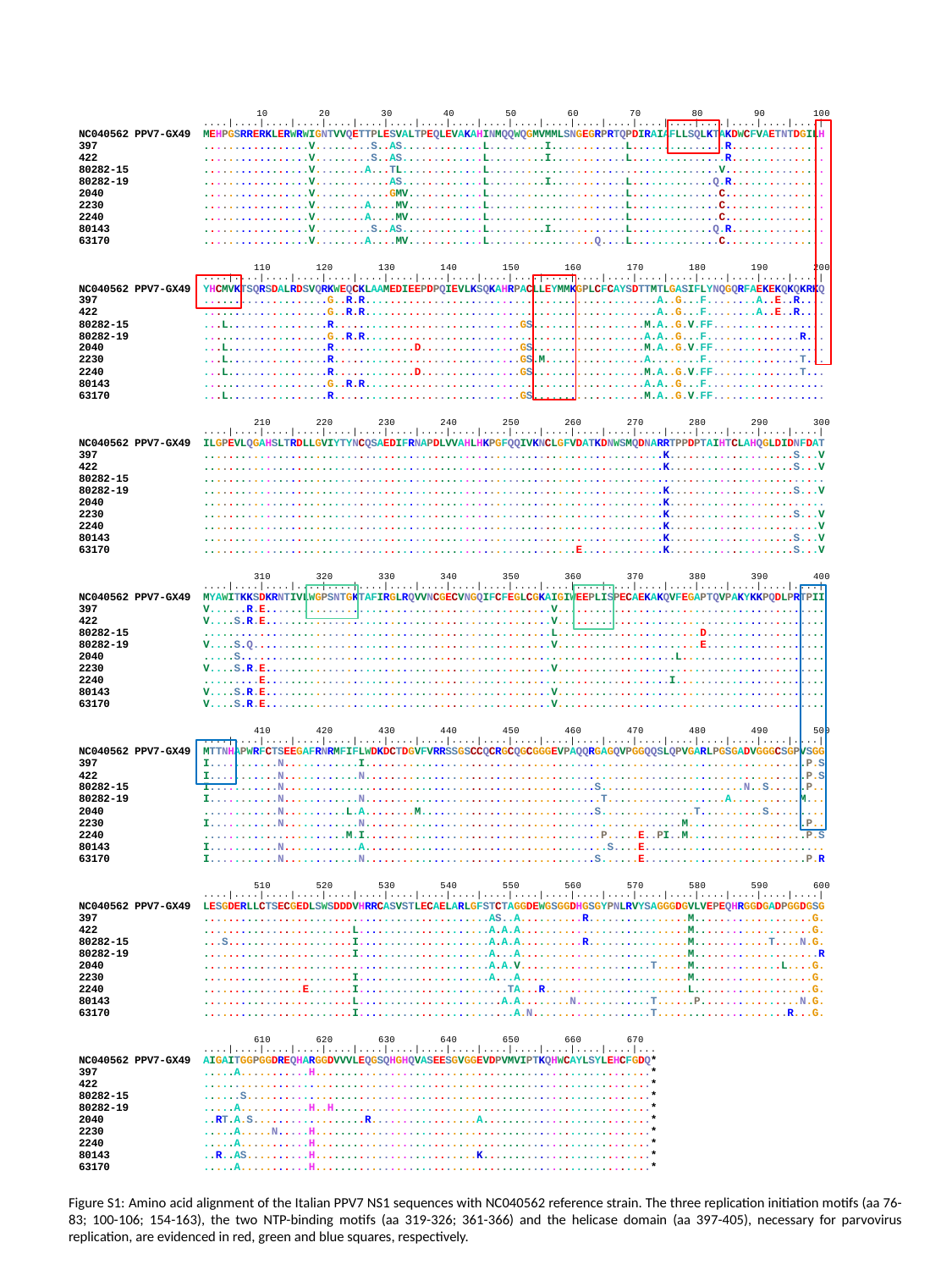

Figure S1: Amino acid alignment of the Italian PPV7 NS1 sequences with NC040562 reference strain. The three replication initiation motifs (aa 76-83; 100-106; 154-163), the two NTP-binding motifs (aa 319-326; 361-366) and the helicase domain (aa 397-405), necessary for parvovirus replication, are evidenced in red, green and blue squares, respectively.
