# Supplementary material for: First Molecular Characterisation of Porcine Parvovirus 7 (PPV7) in Italy
Source: Viruses. 2024 Jun 8;16(6):932. doi: 10.3390/v16060932 (PMC11209580; doi:10.3390/v16060932)
Supplement: Supplementary file 1 [file viruses-16-00932-s001.zip › Figure S2.pdf]

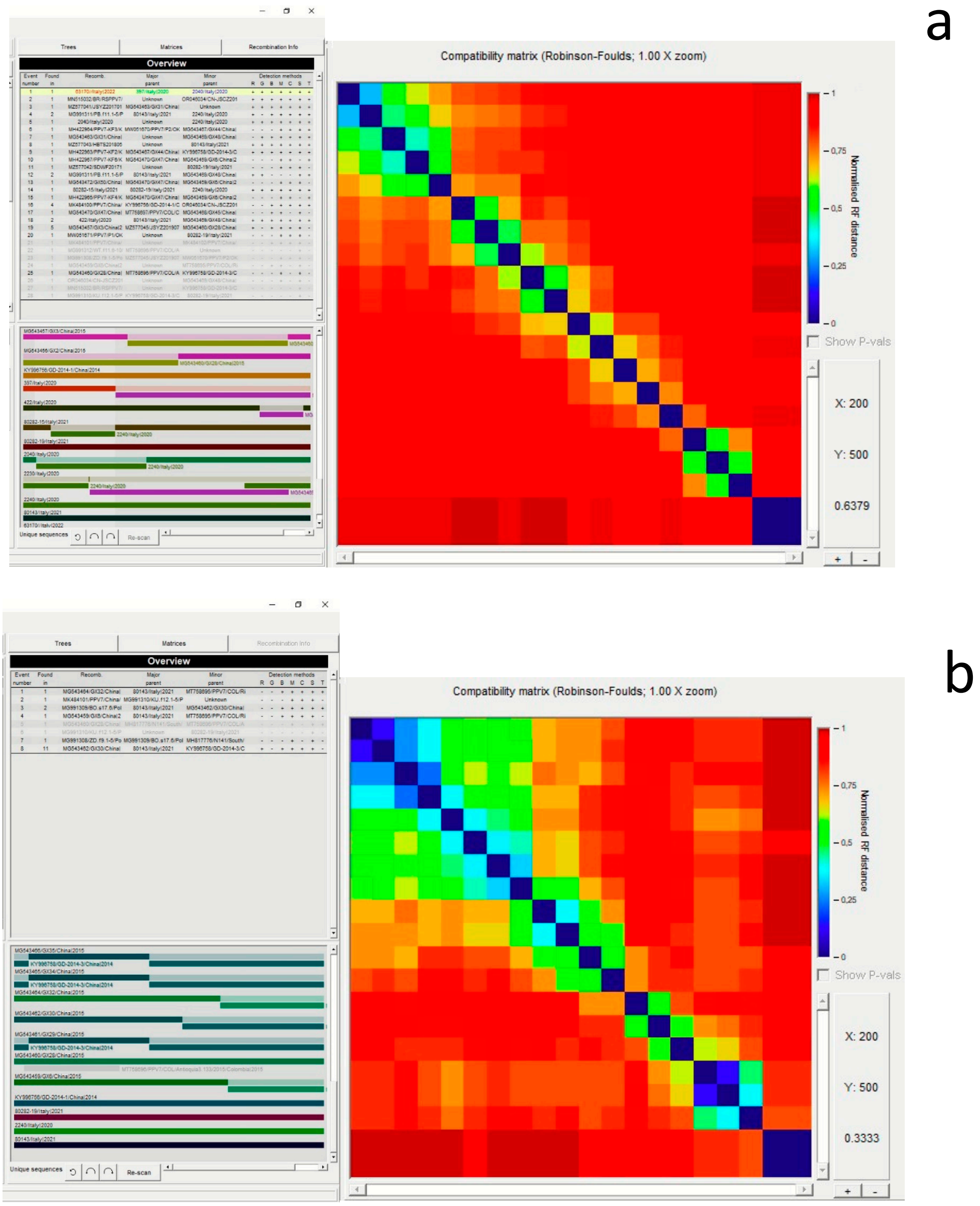
a
Trees
Matrices
Recombination Info
Overview
Compatibility matrix (Robinson-Foulds; 1.00 X zoom)
Normalised RF distance
1
0,75
0,5
0,25
0
Show P-vals
X: 200
Y: 500
0.6379
Unique sequences
Re-scan
b
Trees
Matrices
Recombination Info
Overview
Compatibility matrix (Robinson-Foulds; 1.00 X zoom)
Normalised RF distance
1
0,75
0,5
0,25
0
Show P-vals
X: 200
Y: 500
0.3333
Unique sequences
Re-scan

c

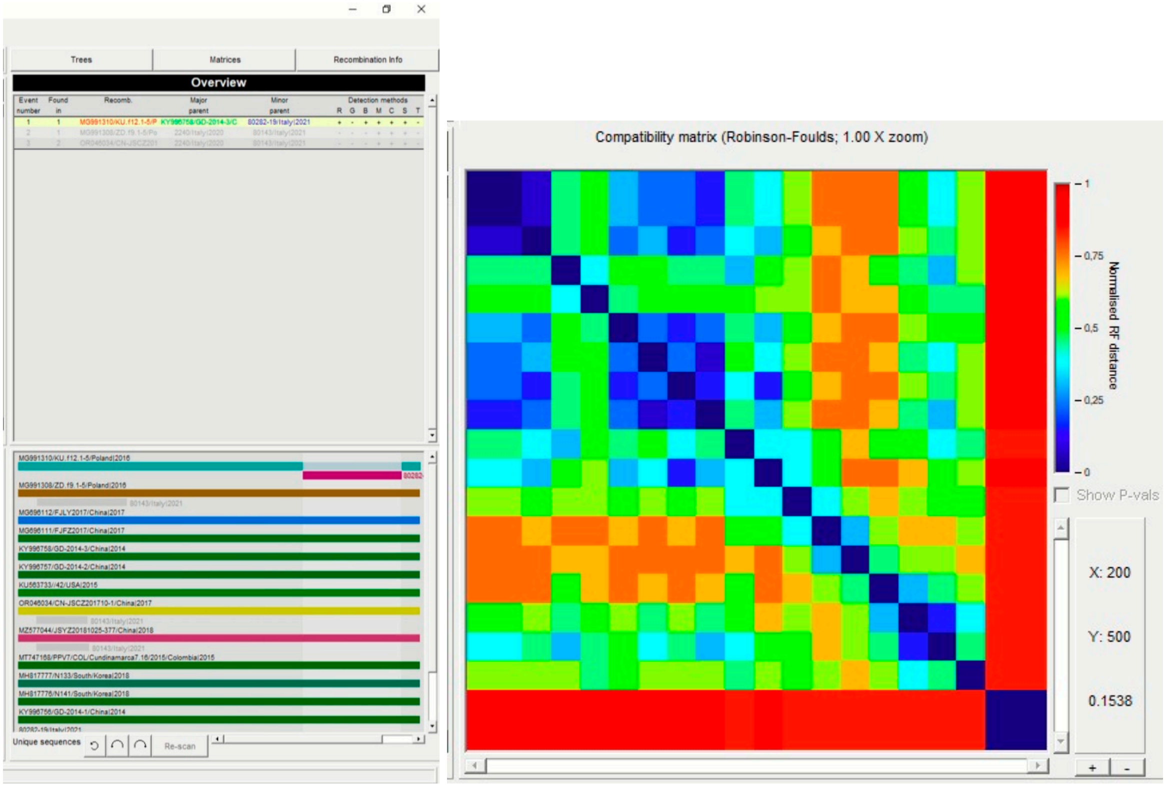

d

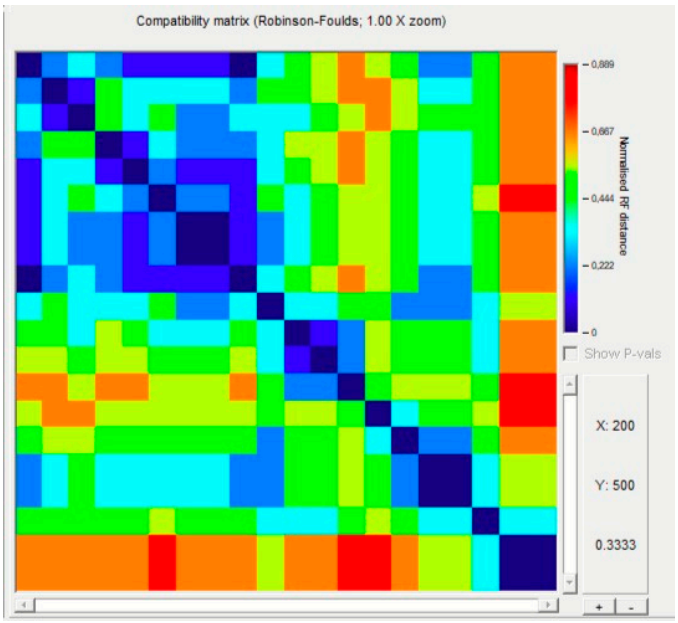

Figure S2: RDP4 output and compatibility matrix (Robinson-Foulds) of the alignment composed of 61 sequences (a), 33 sequences (b), 16 sequences (c). Compatibility matrix of the alignment composed of 12 sequences (d).
