# Supplementary material for: First Molecular Characterisation of Porcine Parvovirus 7 (PPV7) in Italy
Source: Viruses. 2024 Jun 8;16(6):932. doi: 10.3390/v16060932 (PMC11209580; doi:10.3390/v16060932)
Supplement: Supplementary file 1 [file viruses-16-00932-s001.zip › Figure S3.pptx]

## Slide 1
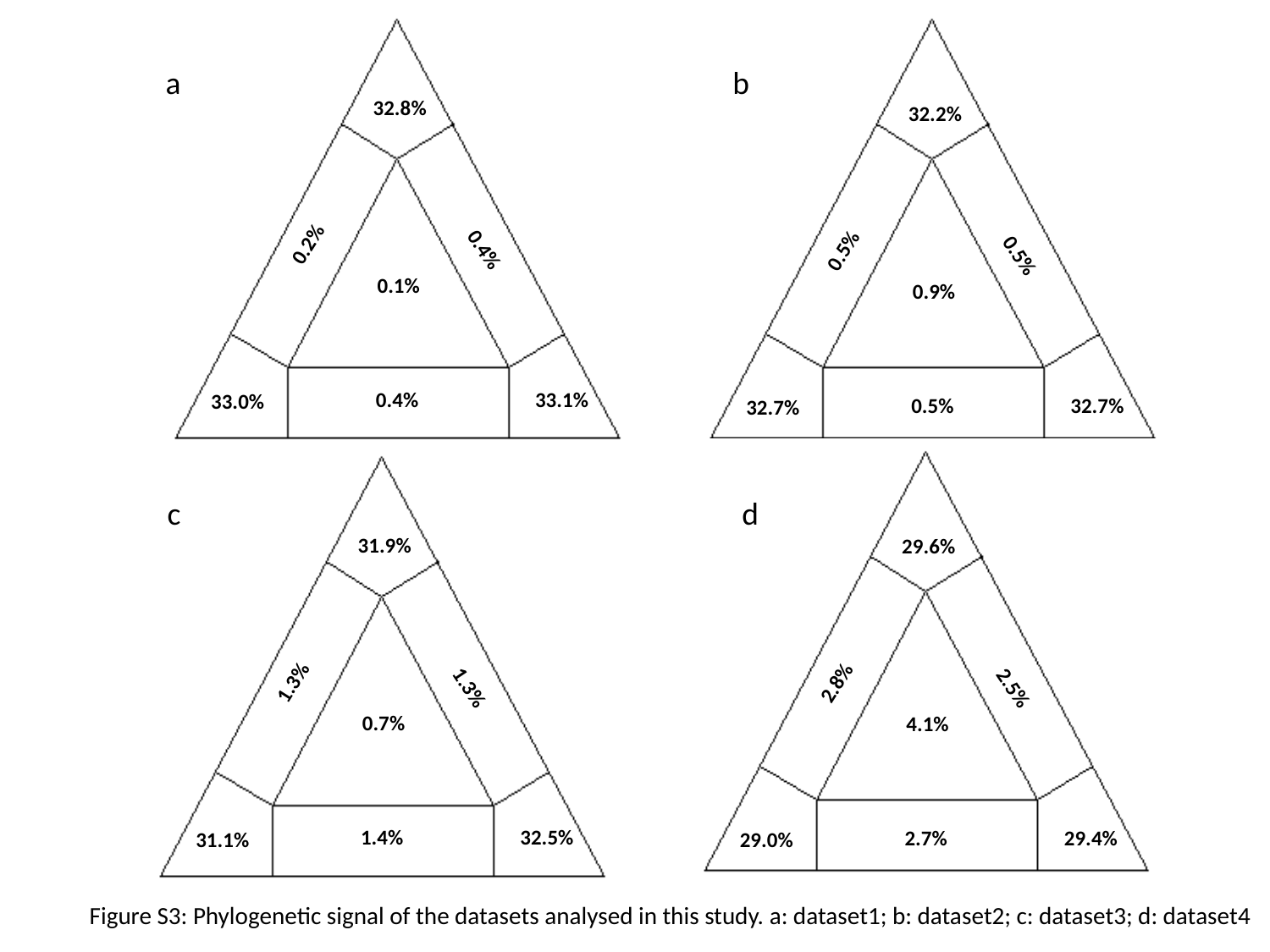

32.8%
0.2%
0.4%
0.1%
33.1%
0.4%
33.0%
32.2%
0.5%
0.5%
0.9%
32.7%
0.5%
32.7%
a
b
29.6%
2.8%
2.5%
4.1%
29.4%
2.7%
29.0%
31.9%
1.3%
1.3%
0.7%
32.5%
1.4%
31.1%
c
d
 Figure S3: Phylogenetic signal of the datasets analysed in this study. a: dataset1; b: dataset2; c: dataset3; d: dataset4
