# Supplementary material for: First Molecular Characterisation of Porcine Parvovirus 7 (PPV7) in Italy
Source: Viruses. 2024 Jun 8;16(6):932. doi: 10.3390/v16060932 (PMC11209580; doi:10.3390/v16060932)
Supplement: Supplementary file 1 [file viruses-16-00932-s001.zip › Figure S4.pptx]

## Slide 1
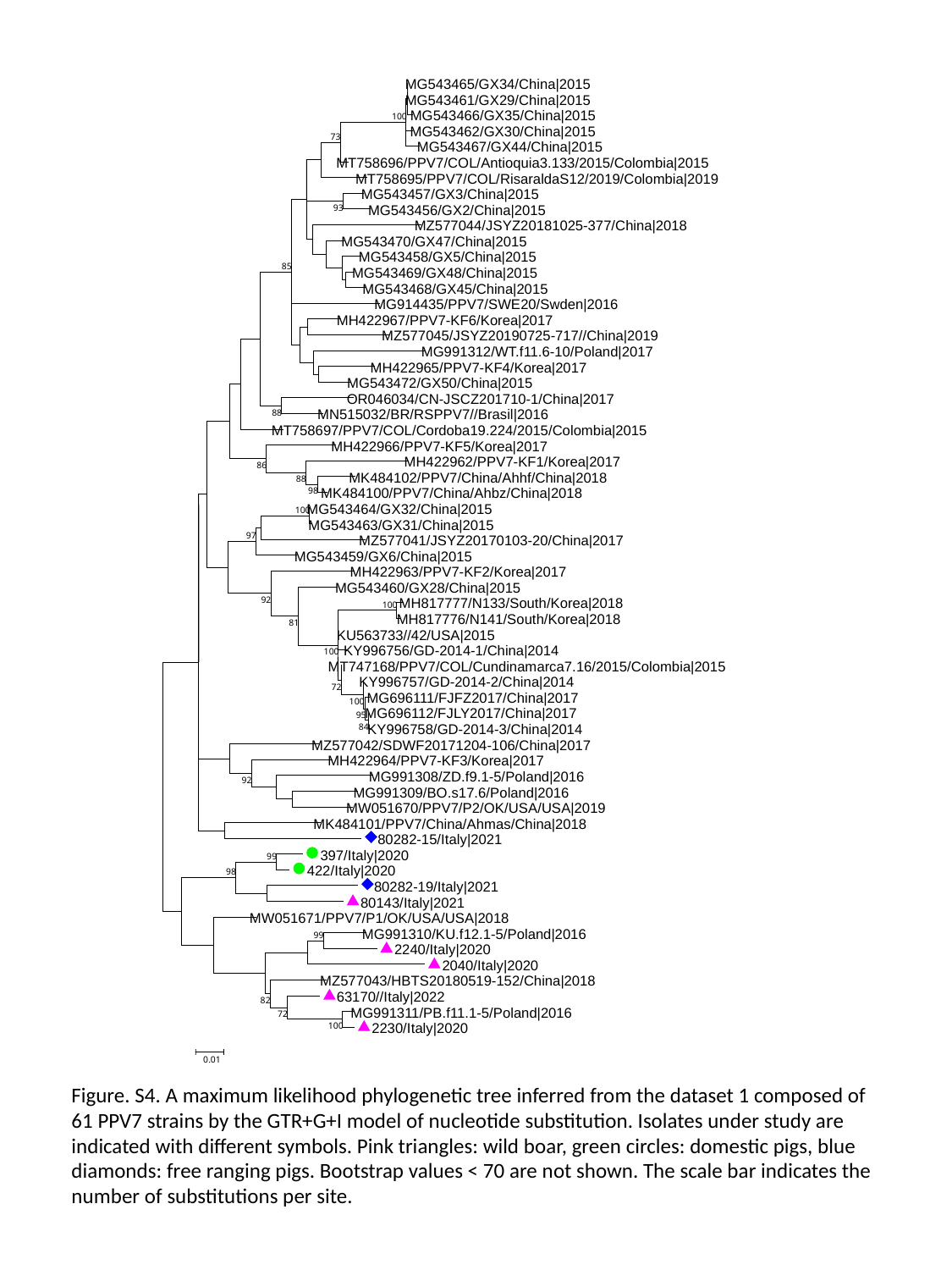

MG543465/GX34/China|2015
 MG543461/GX29/China|2015
 MG543466/GX35/China|2015
100
 MG543462/GX30/China|2015
 MG543467/GX44/China|2015
 MT758696/PPV7/COL/Antioquia3.133/2015/Colombia|2015
 MT758695/PPV7/COL/RisaraldaS12/2019/Colombia|2019
 MG543457/GX3/China|2015
 MG543456/GX2/China|2015
 MZ577044/JSYZ20181025-377/China|2018
 MG543470/GX47/China|2015
 MG543458/GX5/China|2015
 MG543469/GX48/China|2015
 MG543468/GX45/China|2015
 MG914435/PPV7/SWE20/Swden|2016
 MH422967/PPV7-KF6/Korea|2017
 MZ577045/JSYZ20190725-717//China|2019
 MG991312/WT.f11.6-10/Poland|2017
 MH422965/PPV7-KF4/Korea|2017
 MG543472/GX50/China|2015
 OR046034/CN-JSCZ201710-1/China|2017
 MN515032/BR/RSPPV7//Brasil|2016
 MT758697/PPV7/COL/Cordoba19.224/2015/Colombia|2015
 MH422966/PPV7-KF5/Korea|2017
 MH422962/PPV7-KF1/Korea|2017
 MK484102/PPV7/China/Ahhf/China|2018
 MK484100/PPV7/China/Ahbz/China|2018
98
 MG543464/GX32/China|2015
100
 MG543463/GX31/China|2015
 MZ577041/JSYZ20170103-20/China|2017
 MG543459/GX6/China|2015
 MH422963/PPV7-KF2/Korea|2017
 MG543460/GX28/China|2015
 MH817777/N133/South/Korea|2018
100
 MH817776/N141/South/Korea|2018
 KU563733//42/USA|2015
 KY996756/GD-2014-1/China|2014
100
 MT747168/PPV7/COL/Cundinamarca7.16/2015/Colombia|2015
 KY996757/GD-2014-2/China|2014
72
 MG696111/FJFZ2017/China|2017
100
 MG696112/FJLY2017/China|2017
95
 KY996758/GD-2014-3/China|2014
84
 MZ577042/SDWF20171204-106/China|2017
 MH422964/PPV7-KF3/Korea|2017
 MG991308/ZD.f9.1-5/Poland|2016
 MG991309/BO.s17.6/Poland|2016
 MW051670/PPV7/P2/OK/USA/USA|2019
 MK484101/PPV7/China/Ahmas/China|2018
 80282-15/Italy|2021
 397/Italy|2020
99
 422/Italy|2020
 80282-19/Italy|2021
 80143/Italy|2021
 MW051671/PPV7/P1/OK/USA/USA|2018
 MG991310/KU.f12.1-5/Poland|2016
 2240/Italy|2020
 2040/Italy|2020
 MZ577043/HBTS20180519-152/China|2018
 63170//Italy|2022
 MG991311/PB.f11.1-5/Poland|2016
 2230/Italy|2020
100
73
93
85
88
86
88
97
92
81
92
98
99
82
72
0.01
Figure. S4. A maximum likelihood phylogenetic tree inferred from the dataset 1 composed of 61 PPV7 strains by the GTR+G+I model of nucleotide substitution. Isolates under study are indicated with different symbols. Pink triangles: wild boar, green circles: domestic pigs, blue diamonds: free ranging pigs. Bootstrap values < 70 are not shown. The scale bar indicates the number of substitutions per site.
