# Supplementary material for: First Molecular Characterisation of Porcine Parvovirus 7 (PPV7) in Italy
Source: Viruses. 2024 Jun 8;16(6):932. doi: 10.3390/v16060932 (PMC11209580; doi:10.3390/v16060932)
Supplement: Supplementary file 1 [file viruses-16-00932-s001.zip › Figure S5.pdf]

a

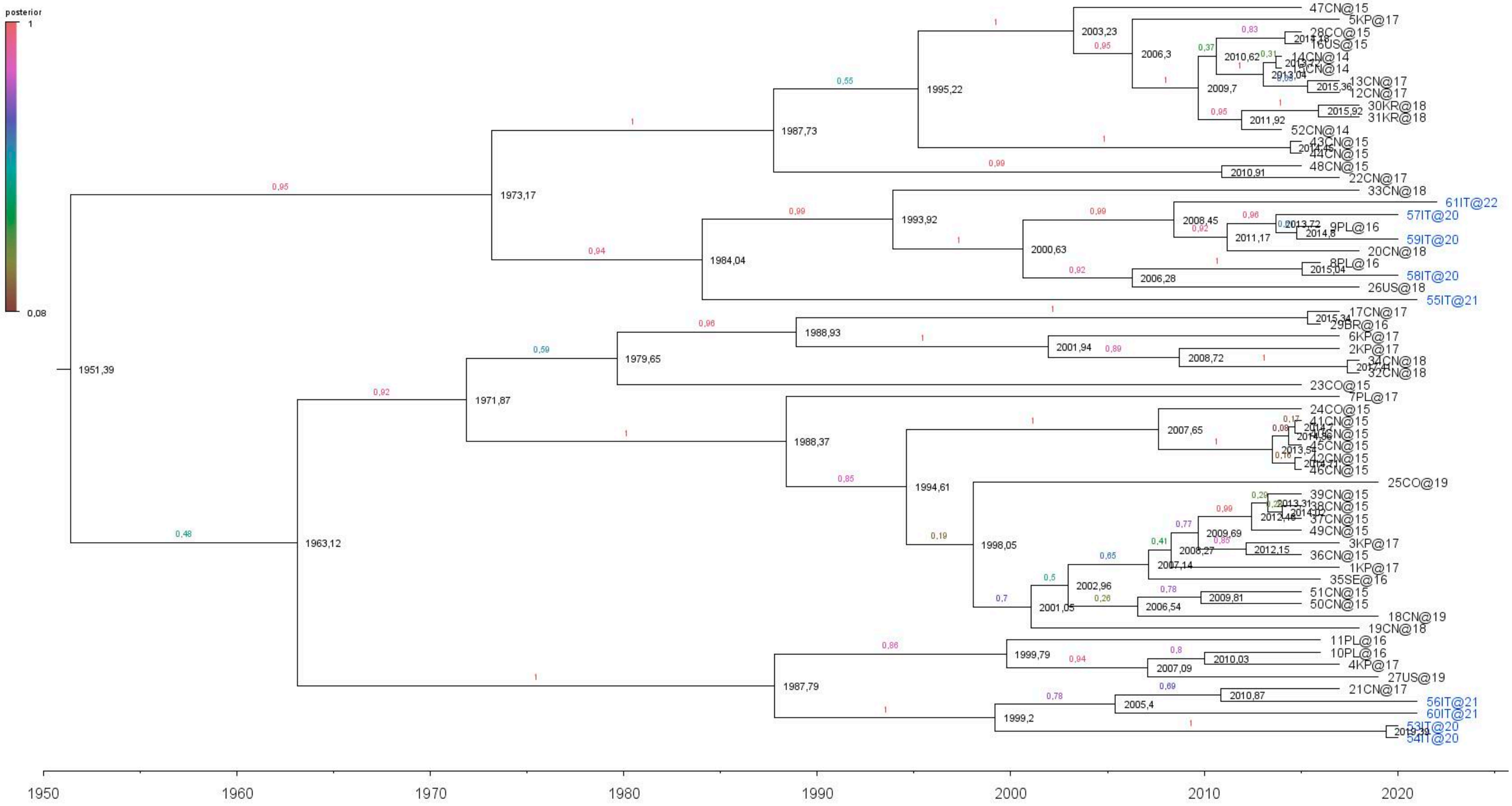

posterior
1
0,08
47CN@15
5KP@17
28CO@15
16US@15
14CN@14
15CN@14
13CN@17
12CN@17
30KR@18
31KR@18
52CN@14
43CN@15
44CN@15
48CN@15
22CN@17
33CN@18
61IT@22
57IT@20
9PL@16
59IT@20
20CN@18
8PL@16
58IT@20
26US@18
55IT@21
17CN@17
29BR@16
6KP@17
2KP@17
34CN@18
32CN@18
23CO@15
7PL@17
24CO@15
41CN@15
45CN@15
42CN@15
46CN@15
25CO@19
39CN@15
38CN@15
37CN@15
49CN@15
3KP@17
36CN@15
1KP@17
35SE@16
51CN@15
50CN@15
18CN@19
19CN@18
11PL@16
10PL@16
4KP@17
27US@19
21CN@17
56IT@21
60IT@21
53IT@20
54IT@20
1951,39
1973,17
1987,73
1995,22
2003,23
2006,3
2010,62
2009,7
2011,92
2010,91
1984,04
1993,92
2000,63
2008,45
2011,17
2006,28
1963,12
1971,87
1979,65
1988,93
2001,94
2008,72
1988,37
1994,61
2007,65
1998,05
2002,96
2001,05
2006,54
2009,81
1987,79
1999,79
2007,09
2010,03
1999,2
2005,4
2010,87
1950
1960
1970
1980
1990
2000
2010
2020

b

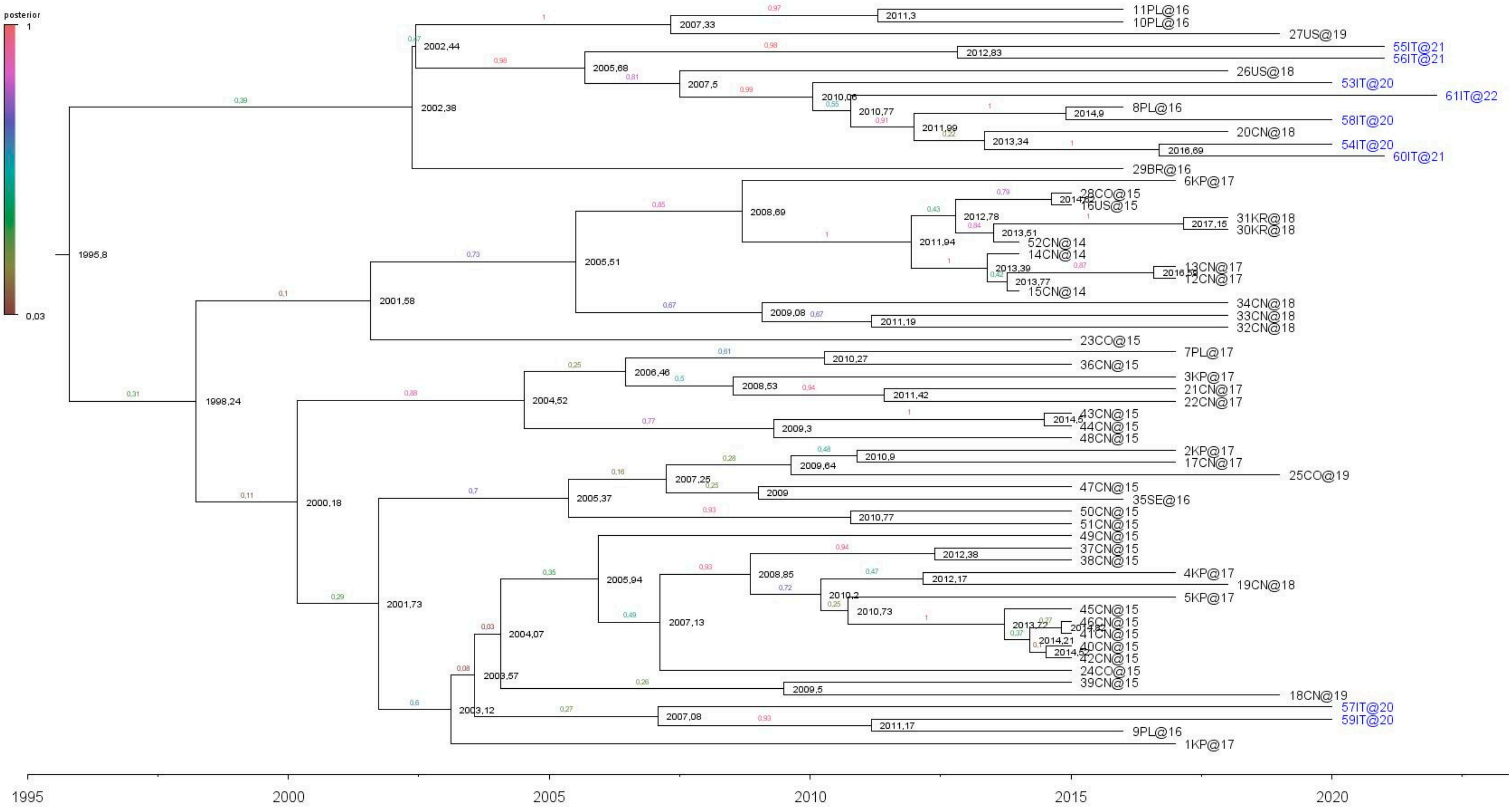
posterior
1
0,03
11PL@16
10PL@16
27US@19
55IT@21
56IT@21
26US@18
53IT@20
61IT@22
8PL@16
58IT@20
20CN@18
54IT@20
60IT@21
29BR@16
6KP@17
28CO@15
16US@15
31KR@18
30KR@18
52CN@14
14CN@14
13CN@17
12CN@17
15CN@14
34CN@18
33CN@18
32CN@18
23CO@15
7PL@17
36CN@15
3KP@17
21CN@17
22CN@17
43CN@15
44CN@15
48CN@15
2KP@17
17CN@17
25CO@19
47CN@15
35SE@16
50CN@15
51CN@15
49CN@15
37CN@15
38CN@15
4KP@17
19CN@18
5KP@17
45CN@15
46CN@15
41CN@15
40CN@15
42CN@15
24CO@15
39CN@15
18CN@19
57IT@20
59IT@20
9PL@16
1KP@17
1995
2000
2005
2010
2015
2020

c

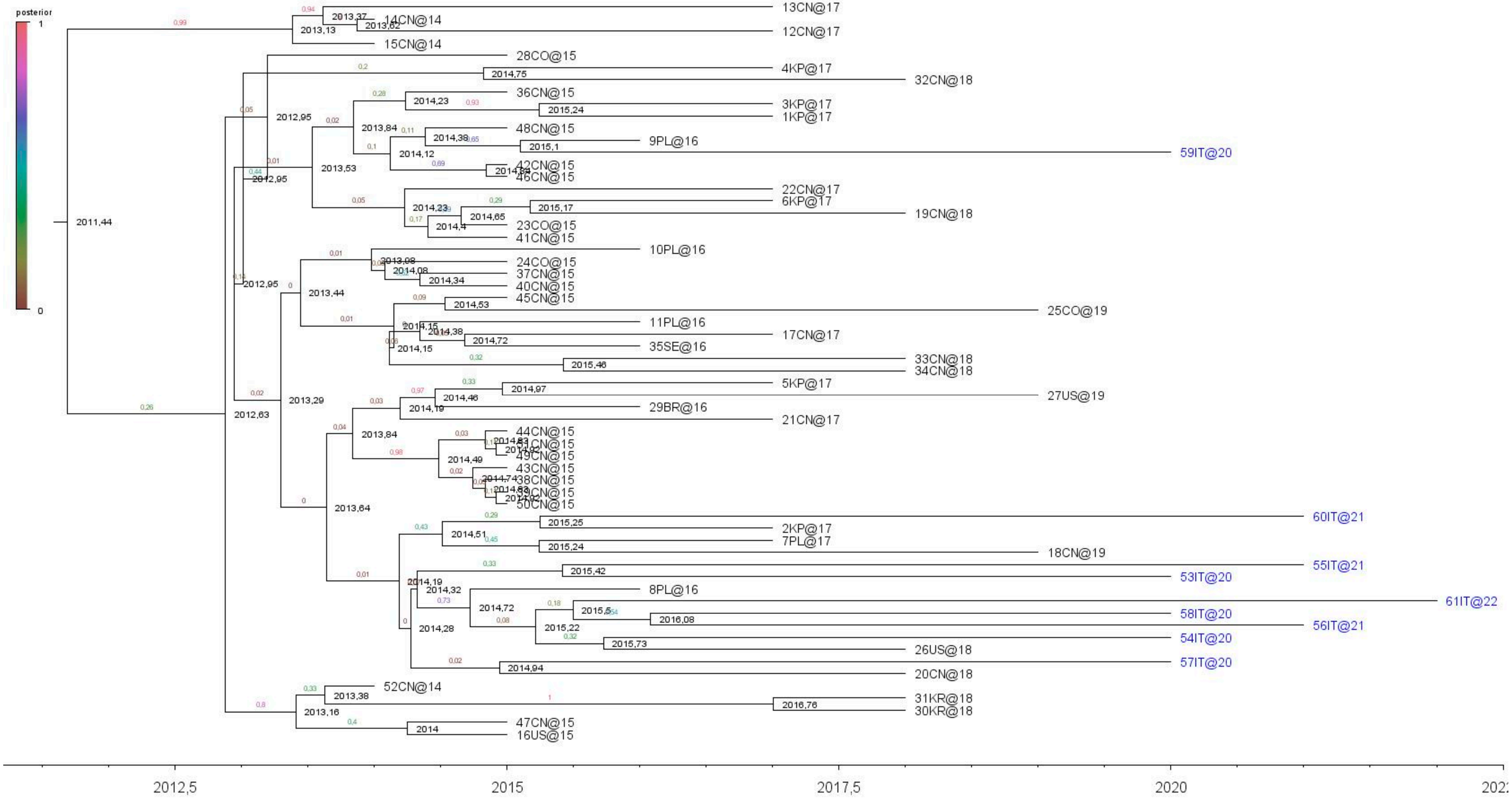

Figure S5. The Bayesian maximum clade credibility tree of the dataset 2 (a), 3 (b), and 4 (c). The node age and the statistical support for the clade (posterior probability) are shown near the nodes and above the branches, respectively. The scale at the bottom of the tree represents time in years.
