# Supplementary material for: First Molecular Characterisation of Porcine Parvovirus 7 (PPV7) in Italy
Source: Viruses. 2024 Jun 8;16(6):932. doi: 10.3390/v16060932 (PMC11209580; doi:10.3390/v16060932)
Supplement: Supplementary file 1 [file viruses-16-00932-s001.zip › Table S1.pdf]

Table S1a. Marginal Likelihood estimates for different molecular clock models of PPV7 datasets2-4 using Path Sampling and Stepping-Stone analysis

Dataset		Molecular clock model		
	Marginal likelihood	Strict	Uncorrelated lognormal relaxed	Random local
Dataset2	Path Sampling	-3941,30	-3932,45	-3943,95
	Stepping-Stone	-3941,43	-3932,41	-3944,27
Dataset3	Path Sampling	-5530,01	-5508,30	-5519,23
	Stepping-Stone	-5530,12	-5508,34	-5519,52
Dataset4	Path Sampling	-3711,93	-3683,71	-3684.12
	Stepping-Stone	-3712,58	-3683,88	-3684,47

Table S1b. Marginal Likelihood estimates for different coalescent models under Uncorrelated lognormal relaxed clock of PPV7 datasets2-4 using Path Sampling and Stepping-Stone analysis

Dataset		Coalescent tree prior		
	Marginal likelihood	Constant population size	Exponential growth	Non-parametric Bayesian Skyline plot
Dataset2	Path Sampling	-3932,45	-3926,68	-3932,94
	Stepping-Stone	-3932,41	-3926,93	-3933,31
Dataset3	Path Sampling	-5508,30	-5488,15	-5489,26
	Stepping-Stone	-5508,34	-5487,78	-5490,05
Dataset4	Path Sampling	-3683,71	-3652,50	-3664,70
	Stepping-Stone	-3683,88	-3652,51	-3665,15
