# Supplementary material for: First Molecular Characterisation of Porcine Parvovirus 7 (PPV7) in Italy
Source: Viruses. 2024 Jun 8;16(6):932. doi: 10.3390/v16060932 (PMC11209580; doi:10.3390/v16060932)
Supplement: Supplementary file 1 [file viruses-16-00932-s001.zip › Table S2.pdf]

DATASET	Identity	Mutation rate	95% HPD Mutation rate	Root date	95%HPD Root date
Dataset2	0,896-1,00	1,18 x 10^{-3}	2,88 x 10^{-4}; 2,12 x 10^{-3}	1951,39	1867,92 - 1993,64
Dataset3	0,900-1,00	3,52 x 10^{-3}	9,32 x 10^{-4}; 6,36 x 10^{-3}	1995,8	1961,35 - 2009,35
Dataset4	0,944-1,00	5,96 x 10^{-3}	3,23 x 10^{-3}; 9,79 x 10^{-3}	2011,44	2008,9 - 2013,3

Table S2: identity, and estimated mutation rate (95%HPD) and root date (95%HPD) for the datasets2-4 analysed in this study.
